# Evaluation of relative biological effectiveness for diseases of the circulatory system based on microdosimetry

**DOI:** 10.1093/jrr/rrae051

**Published:** 2024-06-26

**Authors:** Tatsuhiko Sato, Yusuke Matsuya, Nobuyuki Hamada

**Affiliations:** Nuclear Science and Engineering Center, Japan Atomic Energy Agency (JAEA), Shirakata 2-4, Tokai, Ibaraki 319-1195, Japan; Research Center for Nuclear Physics, Osaka University, Mihogaoka 10-1, Ibaraki, Osaka 567-0047, Japan; Nuclear Science and Engineering Center, Japan Atomic Energy Agency (JAEA), Shirakata 2-4, Tokai, Ibaraki 319-1195, Japan; Faculty of Health Sciences, Hokkaido University, Kita-12 Nishi-5, Kita-ku, Sapporo, Hokkaido 060-0812, Japan; Sustainable System Research Laboratory, Central Research Institute of Electric Power Industry (CRIEPI), Iwado-kita 2-11-1, Komae, Tokyo 201-8511, Japan

**Keywords:** relative biological effectiveness (RBE), microdosimetry, diseases of the circulatory system (DCS), radiological protection, Particle and Heavy Ion Transport code System (PHITS)

## Abstract

In the next decade, the International Commission on Radiological Protection (ICRP) will issue the next set of general recommendations, for which evaluation of relative biological effectiveness (RBE) for various types of tissue reactions would be needed. ICRP has recently classified diseases of the circulatory system (DCS) as a tissue reaction, but has not recommended RBE for DCS. We therefore evaluated the mean and uncertainty of RBE for DCS by applying a microdosimetric kinetic model specialized for RBE estimation of tissue reactions. For this purpose, we analyzed several RBE data for DCS determined by past animal experiments and evaluated the radius of the subnuclear domain best fit to each experiment as a single free parameter included in the model. Our analysis suggested that RBE for DCS tends to be lower than that for skin reactions, and their difference was borderline significant due to large variances of the evaluated parameters. We also found that RBE for DCS following mono-energetic neutron irradiation of the human body is much lower than that for skin reactions, particularly at the thermal energy and around 1 MeV. This tendency is considered attributable not only to the intrinsic difference of neutron RBE between skin reactions and DCS but also to the difference in the contributions of secondary γ-rays to the total absorbed doses between their target organs. These findings will help determine RBE by ICRP for preventing tissue reactions.

## INTRODUCTION

In the latest set of general recommendations [1], the International Commission on Radiological Protection (ICRP) has recommended the dose limits for tissue reactions (formerly termed deterministic effects) in equivalent dose, which is the product of absorbed dose and the radiation weighting factor, *w*_R_. This is based on the assumption that the *w*_R_ values for stochastic effects are higher than the relative biological effectiveness (RBE) for tissue reactions. However, ICRP recently decided to change such equivalent dose limits to absorbed dose limits in the next set of general recommendations [2]. Taken together, the International Commission on Radiation Units and Measurements (ICRU) and ICRP recently suggested that RBE weighting of absorbed dose in relation to the specific health effects could be applied for the protection and operational quantities, as appropriate [3]. Thus, the evaluation of RBE for various types of tissue reactions would be needed to prepare the next ICRP general recommendations to be published in the next decade [4].

To estimate RBE for tissue reactions, we recently proposed a mathematical model applicable to any radiation field [5]. It is based on the microdosimetric kinetic (MK) model [6], which was originally developed for calculating the cell surviving fraction for various types of radiation. Importantly, this model can determine not only the mean value but also the uncertainty of RBE by analysing several experimental data taken from different papers. Using the model, we evaluated the means and uncertainties of RBE for skin reactions and dermal cell survival [5]. It was confirmed that the past evaluation of RBE for tissue reactions made by ICRP [7] and the US National Council on Radiation Protection and Measurements (NCRP) a few decades ago [8] are still supported, the recent experimental data for the skin being taken into account [5].

In this study, we applied this model for evaluating RBE to diseases of the circulatory system (DCS) by analysing animal experimental data taken from several papers [9–13]. We set out to deal with DCS because ICRP recently classified DCS as a tissue reaction with an approximate threshold dose of 0.5 Gy [14], but has not recommended RBE for DCS. The mechanisms of radiation-induced DCS such as the potential target organs/tissues and cellular responses have been intensively discussed [15–19]. In addition, evidence has accumulated for a causal association between radiation exposure and DCS, particularly at high dose [20]. Thus, DCS represents among the most important tissue reactions to be considered in the next ICRP general recommendations.

The basic calculation procedures for evaluating RBE for DCS are the same as those done previously for skin reactions (5). Thus, we here describe procedures briefly, while highlighting the differences between those for skin reactions and DCS. The evaluated RBE values for DCS shall be compared with the corresponding experimental data as well as the previously evaluated RBE values for skin reactions. Then, the appropriate weighting factors for preventing tissue reactions are discussed by calculating the means and uncertainties of RBE for DCS and skin reactions in a human phantom irradiated by mono-energetic neutrons.

## MATERIAL AND METHODS

### Model principles

The original MK model expresses the surviving fraction of cells, *S*(*D*), by the linear-quadratic (LQ) function of absorbed dose *D* [6]. However, the quadratic term of surviving fraction and radiotherapy toxicity is known to become smaller with increasing dose [21–24]. We therefore introduced a certain threshold dose level, *D*_t_, for replacing the linear and LQ functions as written by: 


(1) \begin{align*} S(D)=&\ {S}_{\mathrm{L}\mathrm{Q}}(D)=\exp \left[-\left({\alpha}_0+\beta{z}^{\ast}\right)D-\beta{D}^2\right]\ \mathrm{for}\ D\le{D}_{\mathrm{t}}\nonumber\\{}=&\ {S}_{\mathrm{L}}(D)={S}_{\mathrm{L}\mathrm{Q}}\left({D}_{\mathrm{t}}\right)\exp \left[-{\alpha}_{\mathrm{t}}\left(D-{D}_{\mathrm{t}}\right)\right]\ \mathrm{for}\ D>{D}_{\mathrm{t}}, \end{align*}

where *α*_0_ is the linear coefficient of the surviving fraction with the limit of linear energy transfer (LET) → 0, *β* is the quadratic coefficient independent of radiation quality, and *α*_t_ is the linear coefficient of the surviving fraction at higher doses, satisfying the condition:


(2) \begin{equation*} {\alpha}_{\mathrm{t}}=\left({\alpha}_0+\beta{z}^{\ast}\right)+2\beta{D}_{\mathrm{t}} \end{equation*}

to make *S*_L_(*D*) asymptotic to *S*_LQ_(*D*) at *D*_t_. z^*^ is the saturation-corrected dose-mean specific energy introduced in the MK model by Kase *et al*. [25], which can be determined by


(3) \begin{equation*} {\displaystyle \begin{array}{c}{z}^{\ast }=\frac{1}{\mathrm{\pi} {r}_{\mathrm{d}}^2}{y}^{\ast }=\frac{1}{\mathrm{\pi} {r}_{\mathrm{d}}^2}{y}_0^2\int \frac{\left[1-\exp \left(-{y}^2/{y}_0^2\right)\right]d(y)}{y}\mathrm{d}y,\end{array}} \end{equation*}

where *y* is lineal energy, y^*^ is the saturation-corrected lineal energy, *r*_d_ is the radius of a subnuclear structure referred to as a domain, *y*_0_ is a so-called saturation parameter indicating the lineal energy above which the saturation correction due to the overkill effect becomes very important, and *d*(*y*) is the dose probability density in the domain.

To reduce the number of parameters used in the model, we introduced the relationship:


(4) \begin{equation*} {\displaystyle \begin{array}{c}{\alpha}_0/\beta ={\left(\alpha /\beta \right)}_{\mathrm{c}}-{z}_{\mathrm{c}}^{\ast},\end{array}} \end{equation*}

where (*α*/*β*)_c_ is a constant value of the *α*/*β* ratio evaluated from clinical data, and ${z}_{\mathrm{c}}^{\ast }$ is the saturation-corrected specific energy for a typical clinical radiation field. Then, RBE for test radiation as a function of the reference radiation dose *D* can be calculated by 


(5) \begin{equation*} {\displaystyle \begin{array}{l} RBE(D)={RBE}_{\mathrm{L}\mathrm{Q}}(D)\\[10pt]=\frac{{\left(\alpha /\beta \right)}_{\mathrm{c}}-{z}_{\mathrm{c}}^{\ast }+{z}_{\mathrm{t}\mathrm{est}}^{\ast }+\sqrt{{\left[{\left(\alpha /\beta \right)}_{\mathrm{c}}-{z}_{\mathrm{c}}^{\ast }+{z}_{\mathrm{t}\mathrm{est}}^{\ast}\right]}^2+4\left[{\left(\alpha /\beta \right)}_{\mathrm{c}}-{z}_{\mathrm{c}}^{\ast }+{z}_{\mathrm{ref}}^{\ast }+D\right]D}}{2\left[{\left(\alpha /\beta \right)}_{\mathrm{c}}-{z}_{\mathrm{c}}^{\ast }+{z}_{\mathrm{ref}}^{\ast }+D\right]}\ \\[9pt] {}\begin{array}{c}\ \mathrm{for}\ D\le{D}_{\mathrm{t}}\\[6pt] {}={RBE}_{\mathrm{L}}(D)=\frac{D}{\frac{{\left(\alpha /\beta \right)}_{\mathrm{c}}-{z}_{\mathrm{c}}^{\ast }+{z}_{\mathrm{ref}}^{\ast }+2{D}_{\mathrm{t}}}{{\left(\alpha /\beta \right)}_{\mathrm{c}}-{z}_{\mathrm{c}}^{\ast }+{z}_{\mathrm{t}\mathrm{est}}^{\ast }+2{D}_{\mathrm{t}}/{RBE}_{\mathrm{L}\mathrm{Q}}\left({D}_{\mathrm{t}}\right)}\left(D-{D}_{\mathrm{t}}\right)+\frac{D_{\mathrm{t}}}{RBE_{\mathrm{L}\mathrm{Q}}\left({D}_{\mathrm{t}}\right)}}\ \end{array}\\[22pt] {}\mathrm{for}\ \ D>{D}_{\mathrm{t}}\end{array}} \end{equation*}

where ${z}_{\mathrm{test}}^{\ast }$ and ${z}_{\mathrm{ref}}^{\ast }$ are the saturation-corrected specific energies for the test and reference radiations, respectively.

### Parameter determination for DCS

The parameters *y*_0_ and *r*_d_ in Eq. (3) as well as *D*_t_ and (*α*/*β*)_c_ in Eq. (5) are independent of the radiation field and need to be determined for evaluating the RBE for each tissue reaction. Among them, *y*_0_, *D*_t_ and (*α*/*β*)_c_ for DCS are assumed to be the same as those for skin reactions, which are 100 keV/μm, 7 Gy and 10 Gy, respectively. This assumption allows us to analyze the difference of RBE between DCS and skin reactions based on a significance test of evaluated *r*_d_ values for two diseases. *y*_0_ of 100 keV/μm can be considered adequate irrespective of biological endpoints because it is widely used when expressed in LET. The sensitivity of calculated RBE to *D*_t_ and (*α*/*β*)_c_ was investigated by changing the parameters, although it was non-significant for skin reactions (5).

The domain radius, *r*_d_, was independently determined from the least-square fitting of RBE for DCS measured by several animal experiments [9–13]. Table 1 summarizes the basic features of each experiment. These data were selected from ~450 articles found on PubMed with the search terms RBE and cardiovascular or circulatory system. Selection criteria were that the study employed ≥2 types of radiation, and included sufficient information on the experimental setups.

**Table 1:** Experimental data used in the determination of the best-fit *r*_d_ value for DCS

Reference	Test radiation	Reference radiation	Animal species	Weight of Digimouse (g)	Reaction type	*r*_d_ (μm)
Aarnoudse *et al*. 1977 [9]	15 MeV neutron	X-ray (200 kVp)	Rabbit (chinchilla)	3000	Plaque formation	0.173
Broerse *et al.* 1973 [10]	15 MeV neutron	X-ray (300 kV)	Rat	300	Capillary endothelium	0.233
Hoel *et al.* 2017 [11]	Fission neutron	γ-ray from ^60^Co	Mouse (B6CF1)	24.287^*^	Cardiovascular disease mortality	0.237
Yang et al. 1978 [12]	Fission neutron	γ-ray from ^60^Co	Mouse (B6CF1)	24.287^*^	Ultrastructure of the coronary arteries and aorta	0.227
Yang et al. 1984 [13]	High-energy Ne and Fe ions	X-ray (225 kVp)	Neonatal rat	6	Hemorrhage in brain	0.284
					Mean	0.231
					SD	0.039

^*^Original weight of Digimouse [27]

Prior to the least-square fitting, the saturation-corrected specific energies ${z}_{\mathrm{test}}^{\ast }$, ${z}_{\mathrm{ref}}^{\ast }$ and ${z}_{\mathrm{c}}^{\ast }$ in Eq. (5) must be determined for each experimental condition. They were calculated by the Particle and Heavy Ion Transport code System, PHITS [26], coupled with the mesh-type mouse phantom named Digimouse [27]. Since the target animal size is important to characterize the radiation fields, particularly for neutron exposures [28, 29], the size of Digimouse was adjusted to that of the actual experimental animals by scaling its weight using PHITS-based Application for Radionuclide Dosimetry in Meshes[30]. In the PHITS simulations, the size-adjusted Digimouse was irradiated by the test, reference and typical clinical radiations in the isotropic exposure scenario, and the dose probability densities of lineal energy, *d*(*y*), in the murine whole body were calculated using the updated microdosimetric function [31]. Note that the sizes of the target animals are generally small so that the heterogeneity of the radiation fields inside them can be ignored. In these calculations, we chose 0.25 μm for the domain radius and 10 MV X-rays for the typical clinical radiation, though these choices little affected the final calculation results as discussed in our previous paper [5].

The calculated *d*(*y*) for the test, reference and clinical radiation fields were converted to ${z}_{\mathrm{test}}^{\ast }$, ${z}_{\mathrm{ref}}^{\ast }$ and ${z}_{\mathrm{c}}^{\ast }$, respectively, using Eq. (3), and substituted into Eqs. (4) and (5) for calculating the RBE value in each experimental condition. In these calculations, *r*_d_ is the only free parameter and its numerical value was determined with the least-square fitting by minimizing the square of the differences between the measured and calculated RBE values. Then, the mean and standard deviation (SD) of the domain radii evaluated from each paper, $\overline{r_{\mathrm{d}}}$ and ${\sigma}_{r_{\mathrm{d}}}$, respectively, were calculated and compared with the corresponding data for skin reactions. For comparison, a two-sided unpaired t-test was performed.

### Calculation of RBE for mono-energetic neutron irradiation of human phantom

For discussing the appropriate values to be weighted on the absorbed dose for preventing tissue reactions, the means and uncertainties of RBE for DCS and skin reactions were calculated for mono-energetic neutron exposures to the human body. In this calculation, we performed the PHITS simulations by irradiating neutrons from 1 meV to 10 GeV to the ICRP mesh-type adult male reference phantom [32] under the isotropic exposure scenario and calculated *d*(*y*) in the brain, heart wall, blood (circulating the whole body) and skin of the phantom. The brain and heart wall were selected as the potential targets for cerebrovascular and cardiovascular diseases, respectively. Blood was selected for representing the tissues spread over the whole body. The domain radii were set to $\overline{r_{\mathrm{d}}}$ and $\overline{r_{\mathrm{d}}}\pm{\sigma}_{r_{\mathrm{d}}}$, i.e. 0.231 μm and 0.231 $\pm$ 0.039 μm for DCS and $0.187\ \mathrm{\mu} \mathrm{m}$ and 0.187 $\pm$ 0.052 μm for skin reactions. We also performed similar PHITS simulations for exposure to ^60^Co γ-rays (reference radiation) and 10 MV X-rays (clinical radiation). Then, the mean values and the uncertainties of RBE were deduced from the calculated *d*(*y*) using Eqs. (3)–(5). Note that the domain radius and RBE are anti-correlated, and thus, *d*(*y*) calculated by setting the domain radius to $\overline{r_{\mathrm{d}}}+{\sigma}_{r_{\mathrm{d}}}$ gives the minimum value within the RBE uncertainty.

## RESULTS

Table 1 summarizes the best-fit *r*_d_ obtained from each paper, together with their means and SD. The mean *r*_d_ of 0.231 μm for DCS was higher than 0.187 μm for skin reactions, indicating that RBE for DCS tends to be lower than that for skin reactions in the same radiation field. Their difference was borderline significant based on the two-sided unpaired t-test (*P* = 0.064) due to rather large variances of *r*_d_ best-fit to each experiment.

**Fig. 1:**
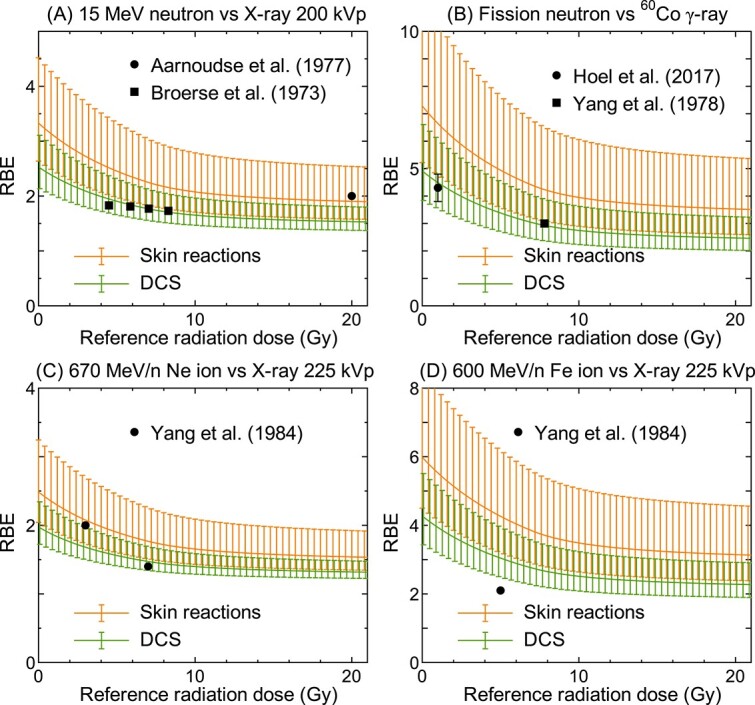
RBE calculated by Eqs. (3)–(5) in comparison with the corresponding experimental data [9–13] as a function of the reference radiation dose. The calculation was performed by setting *r*_d_ to $\overline{r_{\mathrm{d}}}$ and $\overline{r_{\mathrm{d}}}\pm{\sigma}_{r_{\mathrm{d}}}$ for DCS obtained from this study and those for skin reactions determined by our previous study [5]. The reference radiation of the experimental data taken by Broerse *et al.* [10] shown in Panel (A) was 300 kV X-ray instead of 200 kVp X-ray.

Figure 1 compares the measured and calculated RBE for DCS. The calculated results obtained by setting *r*_d_ to $\overline{r_{\mathrm{d}}}\ \mathrm{and}\ \overline{r_{\mathrm{d}}}\pm{\sigma}_{r_{\mathrm{d}}}$ previously evaluate for skin reactions are also shown. The calculated RBE rapidly decreases with increasing dose below ~10 Gy, while becoming asymptotic to certain values at higher dose. This is because the equation for representing the surviving fraction changes from the LQ to linear functions at 7 Gy in our model. The determination of the coefficients, *R*^2^, was 0.88 and − 0.83, respectively, when $\overline{r_{\mathrm{d}}}$ evaluated from DCS and skin reactions were employed.

**Fig. 2:**
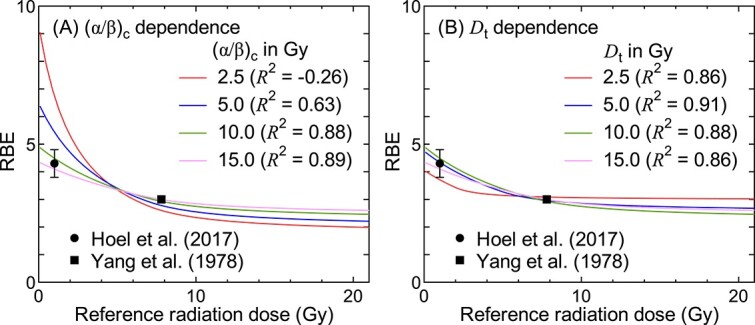
Dependence of the calculated RBE for DCS on (A) (*α*/*β*)_c_ and (B) *D*_t_ for fission neutrons against ^60^Co γ-rays. The experimental data are taken from Refs. [11, 12]. The values in the parentheses are *R*^2^ obtained by analyzing all experimental data listed in Table 1.

Figure 2 shows the dependence of the calculated RBE for DCS on (*α*/*β*)_c_ and *D*_t_. Although RBE data only for fission neutrons against ^60^Co γ-rays are shown in this figure, $\overline{r_{\mathrm{d}}}$ were re-evaluated for each (*α*/*β*)_c_ and *D*_t_ using all experimental data listed in Table 1. Note that *D*_t_ was fixed to 7 Gy when (*α*/*β*)_c_ was the variable, while (*α*/*β*)_c_ was fixed to 10 Gy when *D*_t_ was the variable.

**Fig. 3:**
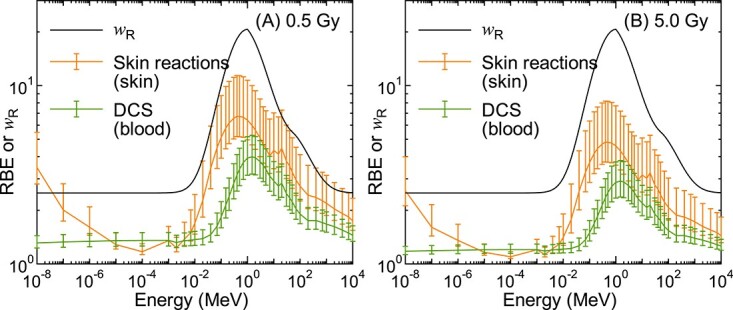
Calculated RBE for DCS and skin reactions for the isotropic irradiation of mono-energetic neutrons to the ICRP mesh-type adult male reference phantom. ^60^Co γ-rays served as the reference radiation with the absorbed doses of (A) 0.5 and (B) 5 Gy in the target organs, which were blood and skin for DCS and skin reactions, respectively, for the data shown in this figure. The radiation weighting factor, *w*_R_, assigned to the neutron is also plotted.

**Fig. 4:**
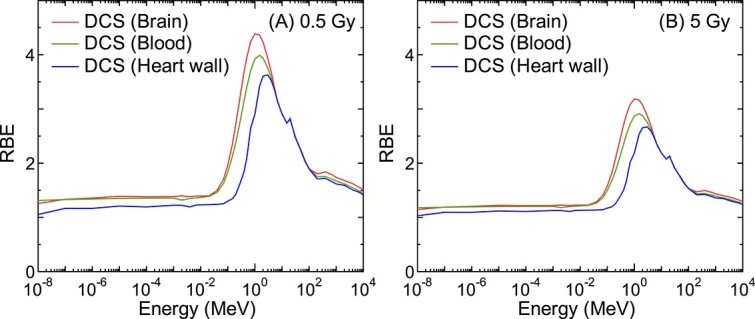
Target-organ dependence of the calculated RBE for DCS for the isotropic irradiation of mono-energetic neutrons to the ICRP mesh-type adult male reference phantom. ^60^Co γ-rays served as the reference radiation with the absorbed doses of (A) 0.5 and (B) 5 Gy in the target organs, which are specified in the parentheses in the legends.

Figure 3 shows the incident-energy dependence of the calculated RBE for DCS and skin reactions for mono-energetic neutron exposures to the ICRP mesh-type adult male reference phantom, together with neutron *w*_R_. Here, ^60^Co γ-rays served as the reference radiation at the absorbed dose of (A) 0.5 and (B) 5 Gy to the target organs (blood for DCS and skin for skin reactions). The uncertainties of RBE estimated by setting *r*_d_ to $\overline{r_{\mathrm{d}}}\pm{\sigma}_{r_{\mathrm{d}}}$ are presented as error bars. Figure 4 shows the target-organ dependence of the calculated RBE for DCS: as expected from Fig. 1, RBE becomes smaller with increasing dose of reference radiation. Note that 0.5 Gy was selected because it is the ICRP threshold for DCS [14], while 5 Gy was selected because this level of dose can be reached during radiotherapy for cancer, e.g. to the heart or aorta following thoracic irradiation [33].

## DISCUSSION

The present model suggests that there is a borderline significant difference between RBE for DCS and skin reactions in the same radiation field. The similarity is probably attributable to common underlying mechanisms, which could be, e.g. inflammation at low dose and cell death at high dose [18]. Given that the mean *r*_d_ for DCS is larger than that for skin reactions, a significant difference could be reached if more experimental data would be available.

As can be seen in Fig. 2, the dose dependence of RBE becomes less significant with increasing (*α*/*β*)_c_ and *D*_t_. The *R*^2^ values are rather insensitive to those parameters except for (*α*/*β*)_c_ below 10 Gy. Such tendency supports our assumption that (*α*/*β*)_c_ and *D*_t_ for DCS are the same as those for skin reactions, i.e. (*α*/*β*)_c_ = 10 Gy and *D*_t_ = 7 Gy, whereas the *α*/*β* ratio of slow turnover tissues such as the heart is considered 2–4 [14].

It is evident from Fig. 3 that the ICRP neutron *w*_R_ for stochastic effects is much larger than the neutron RBE for tissue reactions. The RBE for DCS for mono-energetic neutron irradiation of the human body was also significantly lower than the corresponding RBE for skin reactions, particularly at the thermal energy and around 1 MeV. This tendency is considered attributable not only to the intrinsic difference of neutron RBE between the two diseases but also to the difference in the contributions of secondary γ-rays to the total doses between their target organs, which are generally higher for more deep-seated organs [28, 29]. This dose profile can also explain the difference in RBE between the target organs selected for DCS as shown in Fig. 4; the mean shielding thickness from the body surface is thinner for the brain and thicker for the heart wall than blood. Therefore, the dependence of RBE not only on the absorbed dose but also on the anatomical location of the target organs must be carefully considered in discussing the appropriate weighting factors for preventing tissue reactions including DCS.

The reason for creating the peaks of RBE around 1 MeV is that z^*^ of recoil protons produced by neutron elastic scattering with hydrogen reaches the maximum around the energy. The peak energies are slightly shifted to higher energies with increasing the organ depth because neutrons tend to be moderated before arriving at the target organ. The tiny peaks observed at 20 MeV are mostly attributed to higher cross sections of the ^12^C(n,3α) reaction around the energy. The increase of RBE at the thermal energy observed only for skin reactions is considered attributable to 0.58 MeV protons produced by low-energy neutron capture reaction with nitrogen. Noted that RBE for skin reactions obtained from this study are generally smaller than those calculated in our previous study (5) because neutrons were delivered only to a small piece of skin in their exposure scenario; the γ-ray contributions to the skin dose were less important than the whole body exposure. For example, γ-rays contributed ~58% and 90% of the total dose of the thermal neutron irradiation in the skin and the blood, respectively. The representative exposure scenario should therefore be discussed also in determining the neutron RBE weighting factor for preventing tissue reactions.

## CONCLUSION

The domain radii used in an MK model specialized for RBE estimation of tissue reactions were evaluated by analyzing several RBE data for DCS determined by past animal experiments. The mean domain radius for DCS obtained from this study was larger than the that for skin reaction obtained from the previous study, and the difference was borderline significant due to rather large variances of the evaluated domain radii best fit to each experiment. The means and uncertainties of RBE for DCS and skin reactions were also calculated for mono-energetic neutron irradiation of the human body, using PHITS coupled with the ICRP reference adult male phantom. The calculation results suggest that RBE depends on the anatomical locations of target organs and exposure scenarios, and such dependence should be taken into account in determining the neutron RBE weighting factor for preventing tissue reactions. To get more insights into whether the use of the cell survival model is adequate to estimate RBE for DCS, more RBE data for vascular cell survival would be needed.

## References

[1] International Commission on Radiological Protection . The 2007 recommendations of the international commission on radiological protection. ICRP publication 103. Ann ICRP 2007;37. 10.1016/j.icrp.2007.10.003 18082557

[2] International Commission on Radiological Protection . Use of dose quantities in radiological protection. ICRP publication 147. Ann ICRP 2021;50. 10.1177/0146645320911864. 33653178

[3] International Commission on Radiation Units and Measurements . Operational Quantities for External Radiation Exposure. Bethesda, MD: ICRU Report 95, 2020.

[4] Clement C , Rühm W, Harrison J, et al. Keeping the ICRP recommendations fit for purpose. J Radiol Prot 2021;41:1390–409. 10.1088/1361-6498/ac1611. 34284364

[5] Sato T , Matsuya Y, Hamada N. Microdosimetric modeling of relative biological effectiveness for skin reactions: possible linkage between in vitro and in vivo data. Int J Radiat Oncol Biol Phys 2022;114:153–62. 10.1016/j.ijrobp.2022.05.010. 35589012

[6] Hawkins RB . A microdosimetric-kinetic model of cell death from exposure to ionizing radiation of any LET, with experimental and clinical applications. Int J Radiat Biol 1996;69:739–55. 10.1080/095530096145481. 8691026

[7] International Commission on Radiological Protection . RBE for deterministic effects. ICRP publication 58. Ann ICRP 1990;20. 2699178

[8] National Council on Radiation Protection and Measurements . Radiation Protection Guidance for Activities in Low-Earth Orbit. Bethesda, MD: NCRP Report No. 132, 2000.

[9] Aarnoudse MW , Lamberts HB. Arterial-Wall damage by X-rays and fast-neutrons. Int J Radiat Biol 1977;31:87–94. 10.1080/09553007714550081. 300372

[10] Broerse JJ . Review of RBE values of 15 MeV neutrons for effects on normal tissues. Eur J Cancer 1974;10:225–30. 10.1016/0014-2964(74)90179-0. 4216460

[11] Hoel DG , Carnes BA. Cardiovascular effects of fission neutron or Co-60 exposure in the B6CF(1) mouse. Int J Radiat Biol 2017;93:563–8. 10.1080/09553002.2017.1286051. 28112567

[12] Yang VV , Stearner SP, Ainsworth EJ. Late ultrastructural changes in mouse coronary-arteries and aorta after fission neutron or Co-60-gamma-irradiation. Radiat Res 1978;74:436–56. 10.2307/3574860. 684161

[13] Yang TC , Tobias CA. Effects of heavy ion radiation on the brain vascular system and embryonic development. Adv Space Res 1984;4:239–45. 10.1016/0273-1177(84)90247-3. 11539633

[14] International Commission on Radiological Protection . ICRP statement on tissue reactions / early and late effects of radiation in normal tissues and organs - threshold doses for tissue reactions in a radiation protection context. ICRP publication 118. Ann ICRP 2012;41. 10.1016/j.icrp.2012.02.001. 22925378

[15] Kreuzer M , Auvinen A, Cardis E, et al. Low-dose ionising radiation and cardiovascular diseases - strategies for molecular epidemiological studies in Europe. Mutat Res-Rev Mutat 2015;764:90–100. 10.1016/j.mrrev.2015.03.002. 26041268

[16] Baselet B , Rombouts C, Benotmane AM, et al. Cardiovascular diseases related to ionizing radiation: the risk of low-dose exposure (review). Int J Mol Med 2016;38:1623–41. 10.3892/ijmm.2016.2777. 27748824 PMC5117755

[17] Wang YY , Boerma M, Zhou DH. Ionizing radiation-induced endothelial cell senescence and cardiovascular diseases. Radiat Res 2016;186:153–61. 10.1667/RR14445.1. 27387862 PMC4997805

[18] Tapio S , Little MP, Kaiser JC, et al. Ionizing radiation-induced circulatory and metabolic diseases. Environ Int 2021;146:106235. 10.1016/j.envint.2020.106235. 33157375 PMC10686049

[19] Hamada N . Noncancer effects of ionizing radiation exposure on the eye, the circulatory system and beyond: developments made since the 2011 ICRP statement on tissue reactions. Radiat Res 2023;200:188–216. 10.1667/RADE-23-00030.1. 37410098

[20] Little MP , Azizova TV, Richardson DB, et al. Ionising radiation and cardiovascular disease: systematic review and meta-analysis. Bmj-Brit Med J 2023;380:e072924. 10.1136/bmj-2022-072924. PMC10535030 36889791

[21] Astrahan M . Some implications of linear-quadratic-linear radiation dose-response with regard to hypofractionation. Med Phys 2008;35:4161–72. 10.1118/1.2969065. 18841869

[22] Park C , Papiez L, Zhang S, et al. Universal survival curve and single fraction equivalent dose: useful tools in understanding potency of ablative radiotherapy. Int J Radiat Oncol Biol Phys 2008;70:847–52. 10.1016/j.ijrobp.2007.10.059. 18262098

[23] Elsasser T , Kramer M, Scholz M. Accuracy of the local effect model for the prediction of biologic effects of carbon ion beams in vitro and in vivo. Int J Radiat Oncol Biol Phys 2008;71:866–72. 10.1016/j.ijrobp.2008.02.037. 18430521

[24] Matsuya Y , Kimura T, Date H. Markov chain Monte Carlo analysis for the selection of a cell-killing model under high-dose-rate irradiation. Med Phys 2017;44:5522–32. 10.1002/mp.12508. 28786486

[25] Kase Y , Kanai T, Matsumoto Y, et al. Microdosimetric measurements and estimation of human cell survival for heavy-ion beams. Radiat Res 2006;166:629–38. 10.1667/RR0536.1. 17007551

[26] Sato T , Iwamoto Y, Hashimoto S, et al. Features of particle and heavy ion transport code system PHITS version 3.02. J Nucl Sci Technol 2018;55:684–90. 10.1080/00223131.2017.1419890.

[27] Dogdas B , Stout D, Chatziioannou AF, Leahy RM. Digimouse: a 3D whole body mouse atlas from CT and cryosection data. Phys Med Biol 2007;52:577–87. 10.1088/0031-9155/52/3/003. 17228106 PMC3006167

[28] International Commission on Radiological Protection . Relative biological effectiveness (RBE), quality factor (Q), and radiation weighting factor (w_R_). ICRP publication 92. Ann ICRP 2003;33. 10.1016/S0146-6453(03)00024-1. 14614921

[29] Satoh D , Takahashi F, Endo A, et al. Calculation of dose contributions of electron and charged heavy particles inside phantoms irradiated by monoenergetic neutron. J Radiat Res 2008;49:503–8. 10.1269/jrr.08009. 18580044

[30] Carter LM , Crawford TM, Sato T, et al. PARaDIM: a PHITS-based Monte Carlo tool for internal dosimetry with tetrahedral mesh computational phantoms. J Nucl Med 2019;60:1802–11. 10.2967/jnumed.119.229013. 31201251 PMC6894378

[31] Sato T , Matsuya Y, Ogawa T, et al. Improvement of the hybrid approach between Monte Carlo simulation and analytical function for calculating microdosimetric probability densities in macroscopic matter. Phys Med Biol 2023;68:155005. 10.1088/1361-6560/ace14c. 37352865

[32] International Commission on Radiological Protection . *Adult mesh-type reference computational phantoms*. ICRP publication 145. Ann ICRP 2020;49. 10.1177/0146645319893605. 33231095

[33] McWilliam A , Khalifa J, Osorio EV, et al. Novel methodology to investigate the effect of radiation dose to heart substructures on overall survival. Int J Radiat Oncol Biol Phys 2020;108:1073–81. 10.1016/j.ijrobp.2020.06.031. 32585334

